# Projecting future aboveground carbon sequestration rate of alpine forest on the eastern Tibetan Plateau in response to climate change

**DOI:** 10.3389/fpls.2023.1212406

**Published:** 2023-07-06

**Authors:** Yang Lin, Nan Cong, Jiangtao Xiao, Yongping Kou, Yuanyuan Li, Xinran Yu, Gang Qi, Chaolong Gou, Yongping Bai, Ping Ren

**Affiliations:** ^1^ Key Lab of Land Resources Evaluation and Monitoring in Southwest China, Ministry of Education, Sichuan Normal University, Chengdu, China; ^2^ The Faculty of Geography and Resources Sciences, Sichuan Normal University, Chengdu, China; ^3^ Key Laboratory of Ecosystem Network Observation and Modeling, Lhasa Plateau Ecosystem Research Station, Institute of Geographic Sciences and Natural Resources Research, Chinese Academy of Sciences, Beijing, China; ^4^ Key Laboratory of Mountain Ecological Restoration and Bioresource Utilization & Ecological Restoration and Biodiversity Conservation Key Laboratory of Sichuan Province, Chengdu Institute of Biology, Chinese Academy of Sciences, Chengdu, China; ^5^ Forestry and Grassland Bureau in Mao Country, Aba Tibetan and Qiang Autonomous Prefecture, China

**Keywords:** aboveground carbon sequestration rate, climate change, ecological process model, species and community, Tibetan Plateau, carbon stock

## Abstract

The aboveground carbon sequestration rate (ACSR) of forests serves as an indicator of their carbon sequestration capacity over time, providing insights into the potential carbon sequestration capacity of forest ecosystems. To explore the long-term Spatiotemporal variation of ACSR in the transitional ecotone of the eastern Tibetan Plateau under climate change scenarios, we utilized a forest landscape model that was parameterized with forest inventory data from the eastern Tibetan Plateau to simulate this ecological function changes. The study found that climate warming had significant effect on forests ACSR in different types of forests. ACSR was significantly reduced (p<0.05) in cold temperate coniferous and temperate coniferous forests, whereas it was significantly increased in deciduous broad-leaved forests. However, the impact of climate warming on evergreen broad-leaved forests was found to be negligible. At the species level, climate warming has mostly suppressed the ACSR of coniferous trees, except for Chinese hemlock. The main dominant species, spruce and fir, have been particularly affected. Conversely, the ACSR of most broad-leaved trees has increased due to climate warming. In addition, at the landscape scale, the ACSR within this region is expected to experience a steady decline after 2031s-2036s. Despite the effects of climate warming, this trend is projected to persist. In conclusion, the forests ACSR in this region will be significantly affected by future climate warming. Our research indicates that climate warming will have a noticeable suppressive effect on conifers. It is imperative that this factor be taken into account when devising forest management plans for the future in this region.

## 1 Introduction

Numerous studies have shown that human activities have caused noticeable global warming for decades, which have direct effects on the growth, maturation, and interspecific competition of tree species, changing the productivity, carbon sequestration capacity, and other ecological functions of forests (Lindner et al., 2010; Bachelot et al., 2020; Liu et al., 2021; Liang et al., 2023). The response of forests to future climate remains highly uncertain (Liu et al., 2013; Frank et al., 2015; Schurman et al., 2019). On one hand, high levels of CO_2_ emissions can benefit the growth and productivity of tree species (Franks et al., 2013; Gustafson et al., 2018). However, different tree species exhibit varying responses to elevated CO_2_ concentrations (Kallarackal and Roby, 2012). On the other hand, moderate temperature increases can prolong the growing season and enhance productivity of tree species (Duveneck and Thompson, 2017), but excessive temperature increases can induce heat stress in tree species, resulting in decreased productivity and reduced carbon sequestration capacity (Teskey et al., 2015). This complex process highlights the need for a flexible and suitable indicator to reflect changes in forest carbon sequestration capacity under climate disturbance, the aboveground carbon sequestration rate (ACSR) could reflect the dynamic changes of forest carbon sequestration capacity timely and determine the potential carbon sequestration capacity of forest ecosystems (Chapin et al., 2002; Chen et al., 2013).

Several studies have revealed that climate change will significantly influence the carbon sequestration capacity of forests on a large scale (Ma et al., 2014; Zhou et al., 2022). Yao et al. (2018) introduced a semi-empirical model that demonstrates the positive relationship between rising CO_2_ concentration due to climate change and the increase in total forest biomass and forest carbon sequestration in China. Specifically, transition zone forests have been identified as highly susceptible to the impacts of climate change (Dai et al., 2016; Wu et al., 2020). To fully understand the impact of climate change on forest carbon sequestration capacity, it is crucial to investigate the response of transition zone forests to these changes.

The alpine forests of western Sichuan are the transition zone from the Sichuan Basin to the Tibetan Plateau and are an important ecological barrier in the upper reaches of the Yangtze River, transition from broadleaf-dominated forests of southeast low mountain region to conifers-dominated forest of the northwest alpine region. The transitional ecotone between biomes represents areas where abrupt changes exist in response to climate change (Frelich and Reich, 2010), making this ecotone forest a sensitive region to climate change (Wei and Fang, 2013). In addition, this area has experienced severe harvesting and artificial regeneration (Miao et al., 2014), the forest structure and composition of this region have experienced substantial changes under the interaction of natural restoration and climate change, forest carbon sequestration has also been affected (Huo et al., 2010; Zhang et al., 2023). Therefore, it is necessary to explore the long-term spatial and temporal variation of ACSR in the alpine forests of western Sichuan in the context of climate warming, to develop appropriate forest management strategies for the characteristics of the region.

Assessing the long-term impact of climate change on the carbon sequestration capacity of forests is a challenge, which usually requires a large spatial and temporal scale to be represented, and the long-term complexity of climate change impacts on forest carbon stocks makes changes in forest vegetation carbon with a significant lag (Nevins et al., 2021). Most recent studies on carbon sinks in forest ecosystems on the Tibetan Plateau in response to long-term climate change have been based on model simulations and remote sensing monitoring (Dong and Ni, 2011; Zhao et al., 2014; Sun et al., 2022). However, these models only consider climate change as the main driving force and directly or indirectly ignore forest landscape processes. Whereas forest landscape models provide a favorable tool for studying changes in forest carbon sequestration capacity under climate change at the integrated scale of tree species, community, and landscape over long periods of time, which can not only reflect natural forest succession dynamics, but also set up different disturbance scenarios to help understand the dynamics of forest carbon pools in the context of climate warming through repeated simulation experiments (Shifley et al., 2008; Gustafson et al., 2010).

In this study, we conducted simulation experiments using the LANDIS-II forest landscape model (Scheller et al., 2007), to quantitatively evaluate the temporal and spatial variation characteristics of the ACSR in western Sichuan transitional forests in the next 100 years (2016-2116) under three climate warming scenarios. The aims of this research were to (1) explore the temporal and spatial variation characteristics of the ACSR in alpine forests in western Sichuan under different climate scenarios at various levels, (2) analyze the differences in the impact of different climate scenarios on ACSR using statistical methods, (3) provide scientific insight for selecting appropriate forest management schemes for alpine forests in western Sichuan under the climate warming scenarios. We want to answer these questions to provide useful scientific information for achieving the goals of sustainable forest management to improve the carbon sequestration capacity of the western Sichuan alpine mountains under future climate change.

## 2 Materials and methods

### 2.1 Study area

**Figure 1:**
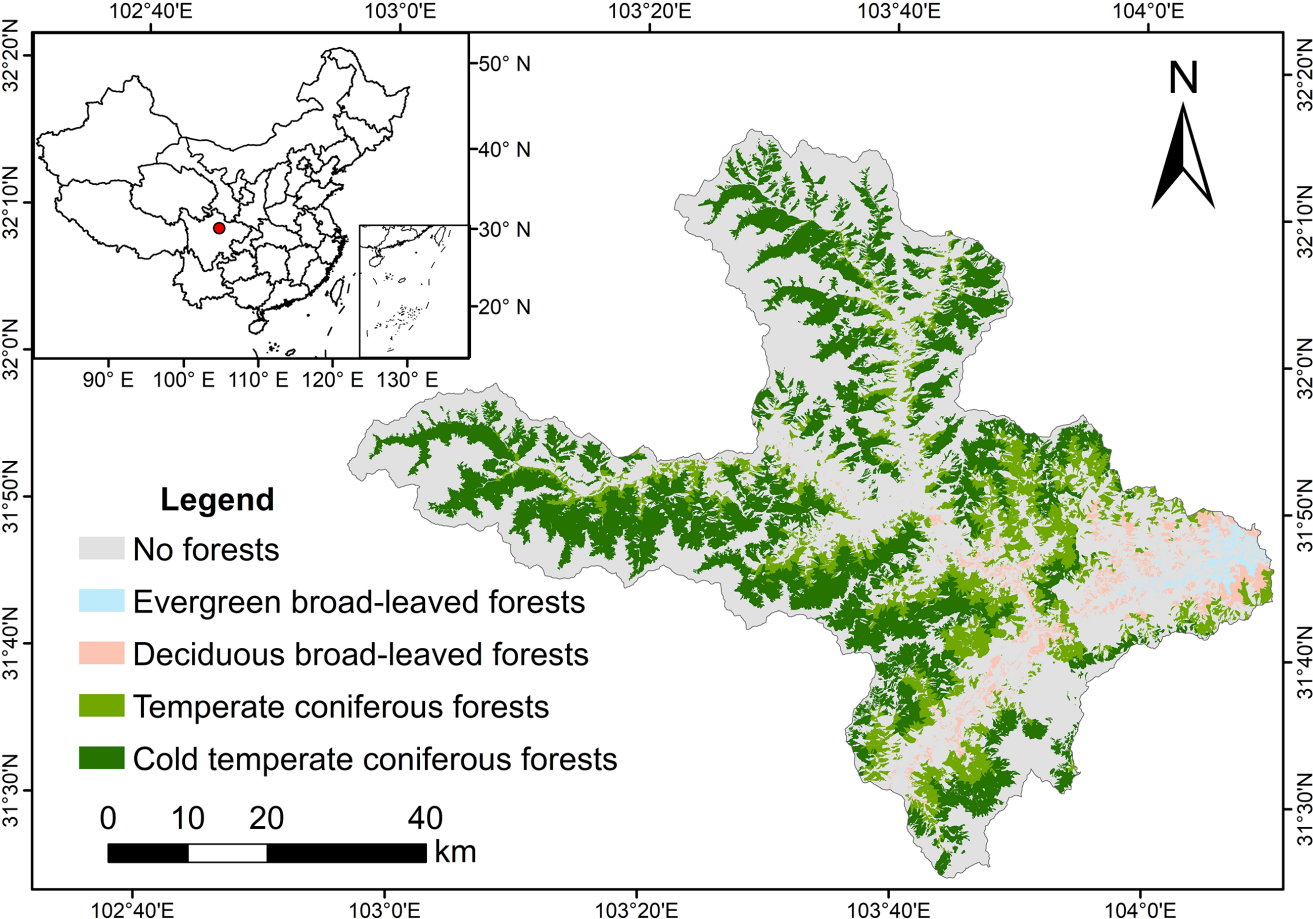
Location and forest landscape types of our simulation.

We conducted our simulation experiment within Mao Country (102°56′~104°10′E, 31°25′~32°16′N)), located in the Western Sichuan Province of Southwestern China (**Figure 1**). The study area encompasses 3903.28 km^2^, with approximately 67.5% of the area covered in forests. The elevation of the region varies greatly, ranging from 890 m to 5230 m, resulting in a diverse vertical and regional climate. The climate in the region is characterized as a highland monsoon, with an average annual temperature of 11.0°C and an annual precipitation of 486.3 mm. The forest landscape of the region is predominantly a mixed forest comprised of both coniferous and broad-leaved forests. The main dominant species in the subalpine region at elevations between 1900 m-3000 m are spruce (*Picea asperata*) and fir (*Abies fabri*). And birch (*Betula* spp.), maple (*Acer* spp.) and ring-cupped oak (*Cyclobalanopsis glauca*) are the main broad-leaved tree species, which are distributed at lower elevations.

The region experienced a large-scale forest harvesting event before 1998, which led to serious damage to its forest ecosystem, and since the implementation of the Natural Forest Protection Project, the forest cover in the region has been restored through afforestation, but due to the homogeneous composition and age structure of planted forests, causing significant changes in forest structure and composition (Zhao and Shao, 2002). At present, the forests in the region are mainly for ecological protection, and forest management is based on natural succession, with no harvesting and afforestation, it can be expected that carbon sequestration capacity will be affected (Piao et al., 2019).

### 2.2 Climate data

To investigate the impact of future climate change on forests ACSR, we considered the current climate and three emission scenarios (RCP2.6, RCP4.5 and RCP8.5) in the experiment (**Supplementary Figure 1**). In the current climate, the monthly mean temperature and precipitation for the study area were obtained from data from 12 surrounding meteorological stations for the period 1980s-2020s and interpolated by regression kriging, and we assumed that the current climate conditions will not change in the next 100 years. In the future climate scenarios, climate datasets that were derived from the Coupled Model Intercomparison Project Phase 5 (CMIP5) framework of the World Climate Research Programme (IPCC, 2013), it is compiled by averaging projections from multiple climate models, which are then reduced and interpolated to a 30-inch grid by the MarkSimGCM weather generator (http://gisweb.ciat.cgiar.org/MarkSimGCM/). CO_2_ concentrations data were from the RCP database (http://tntcat.iiasa), and the photosynthetically active radiation (PAR) data was from the Chinese Ecosystem Research Network Data Center (http://www.nesdc.org.cn/), due to the low changeable rate of PAR (Zhu et al., 2010), we assumed that PAR remains constant in the future scenarios of our study area.

### 2.3 Simulation model

We used a spatially explicit landscape model LANDIS-II v6.0 and an ecological process-based model PnET-II to conduct our research. The LANDIS-II is a stochastic, process-based model that simulates forest development (seed dispersal, tree establishment, growth, competition, mortality) on large spatiotemporal scales (Scheller et al., 2007). Within LANDIS-II model, landscapes are represented as a grid of spatially interacting cells, and each cell is aggregated into different ecological land types according to its site conditions (e.g., climate, soil), forest composition is formed through the use of age cohorts of one or more tree species on each cell, these tree species compete with each other through a variety of vital attributes, ultimately driving successional pathways through competition. And PnET-II is a process-based model that could stimulate the growth of forest trees under specific soil, climate and hydrological conditions (Aber and Federer, 1992), so we used it to calculate the aboveground net primary production (ANPP) and tree species establishment probability (SEP) needed for the LANDIS-II model by simulating the carbon, nitrogen and water cycle processes in the forest.

The key input parameters for the LANDIS-II model include tree species life history parameters, ANPP, SEP and stand spatial parameters (initial tree species distribution raster and ecoregion raster). Life history parameters for 16 dominant tree species were obtained from previous studies and consultation with local forestry experts (Liu et al., 2021) (**Table 1**). The initial distribution raster map of tree species was synthesized by using the forestry survey data of Mao County in 2016. And we divided the study region into five ecoregions based on elevation, land type and soil, including an inactive ecoregion and four active ecoregions, the inactive ecoregion was no-forested and shrubs, the four ecoregions were classified mainly based on elevation, with ecoregion 1 being above 1500 m, ecoregion 2 ranging from 1500 to 2000 m, ecoregion 3 ranging from 2000 to 2800 m, and ecoregion 4 being above 2800 m (**Supplementary Figure 2**). We uniformly set the resolution of the spatial data to 100 m×100 m.

**Table 1:** Life-history attributes of the main tree species in the study area.

Species	Common name	LONG	MTR	ST	ESD	MSD	VSR
*Picea asperata*	Spruce	300	60	4	50	150	0
Abies *fabri*	Fir	300	60	4	50	150	0
*Tsuga chinensis*	Chinese hemlock	400	80	4	100	150	0
*Pinus armandii*	Huashan pine	200	35	2	30	100	0
*Pinus tabuliformis*	Chinese pine	150	35	2	30	100	0
*Quercus wutaishanica*	Liaodong oak	300	40	2	50	200	1
*Quercus semicarpifolia*	Brown oak	250	40	2	50	200	1
*Quercus variabilis*	Cork oak	200	20	3	50	300	0.8
*Cyclobalanopsis glauca*	Ring-cupped oak	250	20	2	50	200	0.9
*Cupressus chengiana*	Minjiang cypress	300	30	3	200	500	0
*Sophora japonica*	Locust	150	15	1	300	1500	0.9
*Toona sinensis*	Chinese toon	120	15	1	300	1000	0.9
*Acer* spp.	Maple	200	20	3	50	200	0.5
*Betula* spp.	Birch	150	15	1	200	1500	0.8
*Populus* spp.	Aspen	150	15	1	300	1500	0.9
*Alnus cremastogyne*	Alder	150	15	2	200	1000	0.8

LONG, longevity (years); MTR, age of maturity; ST, shade tolerance (1-5), 1 represents minimal shade tolerance, and 5 represents maximum shade tolerance; ESD, effective seeding distance; MSD, maximum seeding distance; VSR, vegetative reproduction probability (0-1).

The parameters selected for the PnET-II include climate data (monthly temperature, monthly mean precipitation, PAR and CO_2_ concentration), species physiological parameters (**Supplementary Table 1**), and site conditions parameters. The species physiological parameters reflect the differentiation in the response of different tree species to climate and environmental changes, and the physiological parameters of each tree species are mainly from the relevant literature review and site survey (Duan et al., 2014; Wang et al., 2017; Cai et al., 2019). Latitude and water holding capacity (WHC) are the main parameters in the site conditions, water holding capacity was calculated by rock fragment, clay and sand content obtained from a national soil database (http://gis.soil.csdb.cn/).

The applicability of the LANDIS-II model has been well demonstrated in previous studies (Xu et al., 2009; Gustafson et al., 2010), but the lack of long-term observations on large spatial and temporal scales makes it difficult to visually validate the spatial model. In this study, we compared the initial forest simulation data of LANDIS-II with actual observations (**Supplementary Figure 3**). The comparison results show that the model can better simulate the forest dynamics in Mao County.

### 2.4 Data analysis

To compare the differences in forest carbon sequestration capacity over the next 100 years under different climate scenarios, we calculated aboveground carbon sequestration rates from 2016 to 2116 at the tree species-, community-, and landscape-scales (Fang et al., 2001). ACSR was calculated using the following equation:


$$CSR_{i}=\left(C_{i,t1}-C_{i,t2}\right)/\left(t_{2}-t_{1}\right)$$

where $CSR_{i}$ is the ACSR at a certain period, $C_{i,t1}$ is the biomass at the time $t_{1}$ and $C_{i,t2}$ is the biomass at the time $t_{2}$ .

To visualize the spatial and temporal variation of forest carbon sequestration capacity, we produced spatial maps of aboveground carbon stocks for each cell in the study region. Five iterations of each climate scenario were simulated to reduce the stochasticity of the models. We tested differences in ACSR across climate scenarios by one-way ANOVA with multiple comparisons using LSD, and different letters representing significant differences (P<0.05).

## 3 Results

### 3.1 ACSR at landscape level

**Figure 2:**
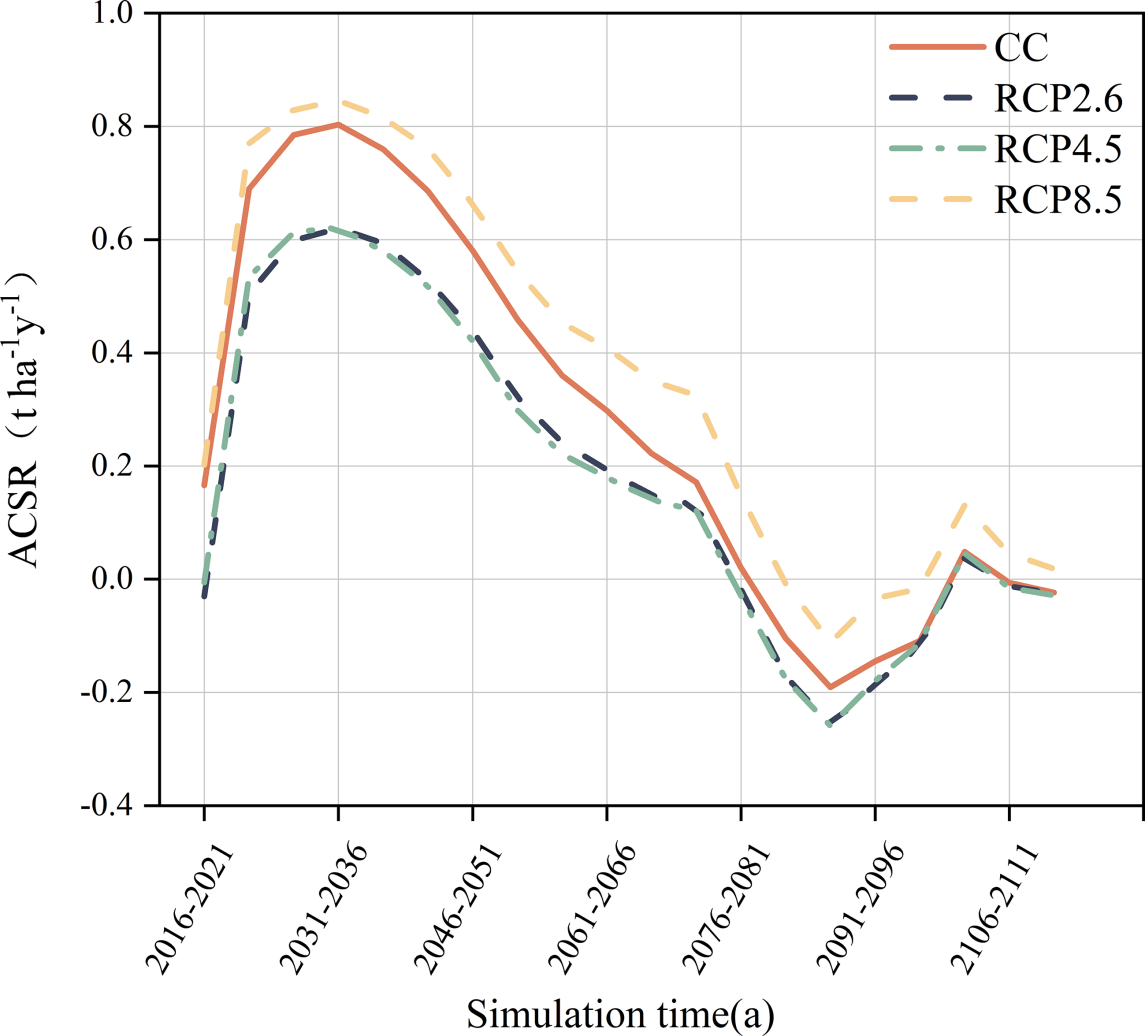
Forest aboveground carbon sequestration rate in landscape level.

ACSR of forests under different climate scenarios varied noticeably in the next 100 years (**Figure 2**). The overall trend in ACSR was consistent over the simulation period under different climate scenarios, with a continuous increase in ACSR from 2016s to 2036s, followed by a persistent decrease in ACSR over the next 50 years, with a short period of fluctuation by the end of the simulation. The overall ACSR was maximum under the RCP8.5 scenario with a mean value of 0.36 t ha^-1^ a^-1^, the mean ACSR under the current climate scenario was 0.27 t ha^-1^ a^-1^, which was slightly lower than the RCP8.5 scenario. The ACSR was minimum in the RCP2.6 and RCP4.5 scenarios, and the difference between them was minor, with mean values of 0.18 t ha^-1^ a^-1^ and 0.17 t ha^-1^ a^-1^, respectively.

### 3.2 ACSR at community level

**Figure 3:**
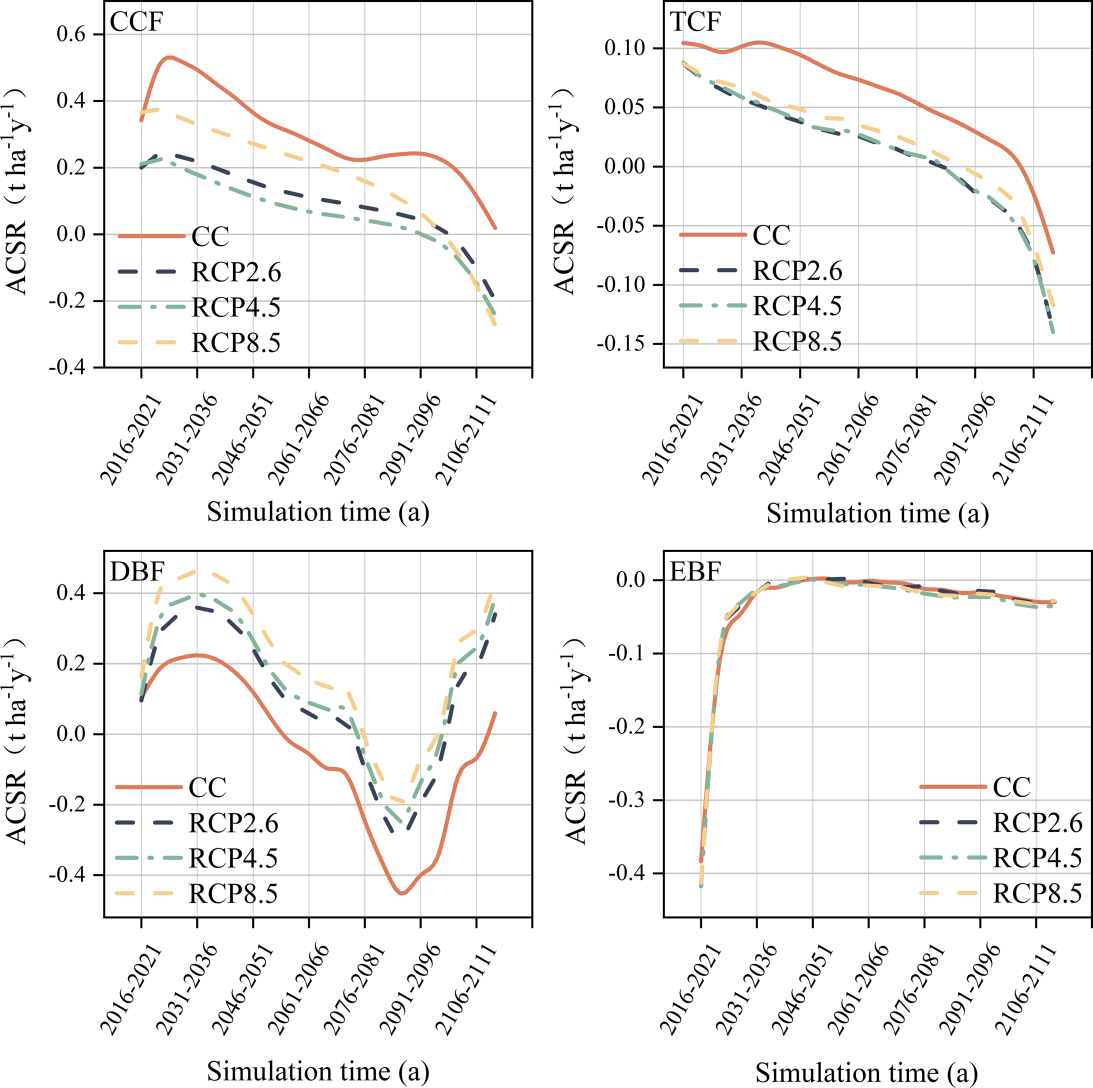
Forest aboveground carbon sequestration rate of different communities. CCF, Cold temperate coniferous forest; TCF, Temperate coniferous forests; DBF, Deciduous broad-leaved forest; EBF, Evergreen broad-leaved forest.

Four forest communities with varying ACSR were simulated under different climate scenarios (**Figure 3**). The ACSR exhibited an overall decreasing trend in all climate scenarios in cold temperate coniferous forests. Furthermore, the forest ACSR was suppressed in the climate warming scenario, particularly in the late simulation period, with negative ACSR observed in all climate warming scenarios. The ACSR of temperate coniferous forests continued to decline under all climate scenarios during the simulation period, and warming also harmed the ACSR of temperate coniferous forests, with the ACSR under all warming scenarios was always lower that than current climate. The ACSR of deciduous broad-leaved forests showed a “v” shape for all climate scenarios during the simulation period, with the minimum values occurring in the 2081s-2086s. And climate warming had a positive effect on the ACSR of deciduous broad-leaved forests, as temperatures increased, this effect became more pronounced, with the highest ACSR observed under the RCP8.5 scenario. The impact of climate warming on the ACSR of evergreen broad-leaved forests was found to be negligible. The ACSR of evergreen broad-leaved forests showed a noticeable increase from 2016s-2036s, regardless of the climate scenario, during this period, the ACSR values shifted from negative to almost 0, eventually stabilizing at around 0.

### 3.3 ACSR at species level

**Figure 4:**
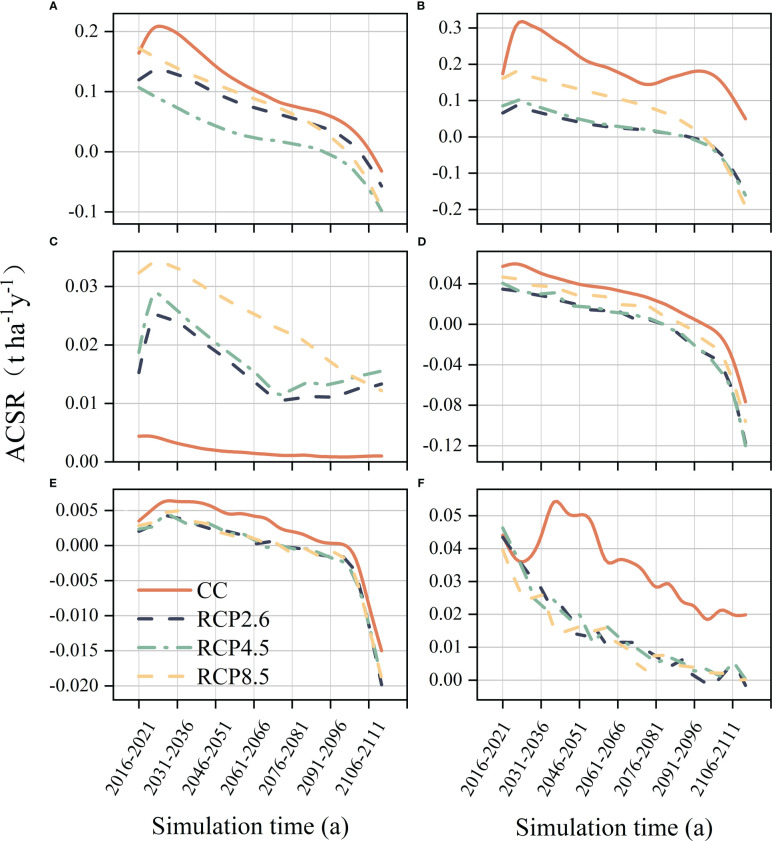
Forest aboveground carbon sequestration rate of coniferous species trees. **(A)** Spruce, **(B)** Fir, **(C)** Chinese hemlock, **(D)** Huashan pine, **(E)** Chinese pine, **(F)** Minjiang cypress.

The simulated response of coniferous species ACSR to climate change in the next 100 years was roughly the similar excluding Chinese hemlock (**Figure 4**). Our study found that climate warming had a negative impact on ACSR in the majority of coniferous forests in the study area. However, it did not alter the overall trend of ACSR in tree species. Like the spruce (**Figure 4A**), the ACSR generally displayed a consistent downward trend throughout the simulation across all climate scenarios, furthermore, its ACSR became negative towards the end of the simulation, with this trend being further amplified by warming. The impact of climate warming on fir was significant, as shown in **Figure 4B**, the ACSR experienced a fluctuating decline under the current climate scenario, although the decline was relatively minor, during the simulation period, the ACSR changed from 0.17 t ha^-1^ a^-1^ to 0.05 t ha^-1^ a^-1^, and this change was exacerbated by climate warming. According to the RCP8.5 scenario, the ACSR decreased from 0.16 t ha^-1^ a^-1^ to -0.19 t ha^-1^ a^-1^, similarly, under the RCP4.5 scenario, it decreased from 0.085 t ha^-1^ a^-1^ to -0.16 t ha^-1^ a^-1^, and under the RCP2.6 scenario, it decreased from 0.066 t ha^-1^ a^-1^ to -0.15 t ha^-1^ a^-1^. Warming had a positive effect on the ACSR of Chinese hemlock (**Figure 4C**), and increased its ACSR substantially throughout the simulation period, with the most pronounced promotion by RCP8.5. ACSR of Huashan pine, Chinese pine and Minjiang cypress has been negatively affected by climate warming, which has exacerbated the decline of their ACSR (**Figures 4D–F**).

**Figure 5:**
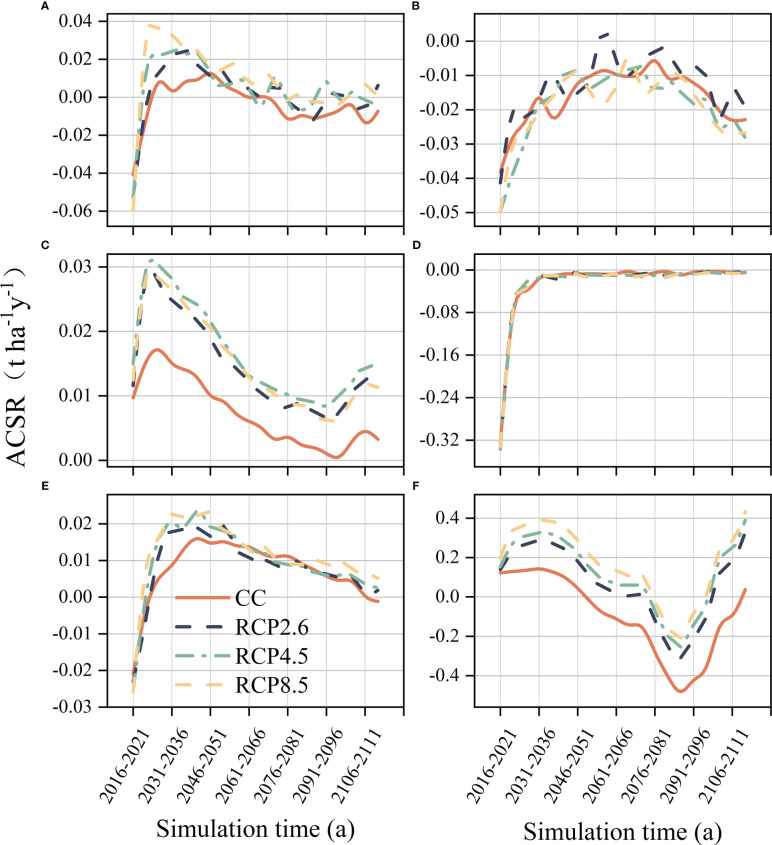
Forest aboveground carbon sequestration rate of broad-leaved species trees. **(A)** Liaodong oak, **(B)** Brown oak, **(C)** Cork oak, **(D)** Ring-cupped oak, **(E)** Maple, **(F)** Birch.

The simulation showed that broad-leaved trees had complex dynamics, as depicted in **Figure 5**. Additionally, the study found that climate warming had a positive impact on the ACSR of the majority of broad-leaved tree species. The ACSR of Liaodong oak exhibited an initial increase followed by a subsequent decrease, specifically, between 2016s and 2036s, the ACSR demonstrated a rapid increase before gradually declining, climate warming does not alter this trend, but enhances the ACSR to some extent when compared to current climate scenario. Throughout the simulation period, warming shifted the mean ACSR of Liaodong oak from a negative value under the current climate to a positive value. The impact of climate warming on brown oak and ring-cupped oak was relatively minor, and the effect of warming on their ACSR was not noticeable. During the simulation period, the ACSR of cork oak exhibited a trend of increasing and then decreasing. The impact of warming on the ACSR was considerable, particularly between 2021s and 2026s, resulting in a substantial increase in the peak ACSR of cork oak. The effect of warming on maple was similar to that on Liaodong oak. The variation of birch’s ACSR resulted in a “v” shape, with a decrease followed by an increase, and the minimum value was observed between 2081s and 2086s. The ACSR of birch was pronouncedly increased due to climate warming, and the most evident positive effect was observed in the RCP8.5 scenario, the simulation revealed that the maximum ACSR can reach 0.43 t ha^-1^ a^-1^.

### 3.4 Differences of ACSR among climate scenarios

**Figure 6:**
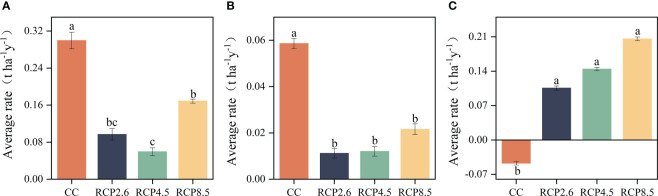
Results of multiple comparisons of the influences on the aboveground carbon sequestration rates of the communities. **(A)** Cold temperate coniferous forests, **(B)** Temperate coniferous forests, **(C)** Deciduous broad-leaved forests. Significant differences between different climate scenarios are indicated with different lowercase letters (P < 0.05).

The results of ANOVA indicated significant differences in ACSR between cold temperate coniferous forests, temperate coniferous forests and deciduous broad-leafed forests under different climate scenarios (p<0.05) (**Figure 6**), and there was no significant effect of climate change on ACSR in evergreen broad-leaved forests. The ACSR of cold temperate coniferous forests has been significantly reduced due to climate warming, with the RCP4.5 scenario showing a significant difference from the other two warming scenarios (**Figure 6A**). Climate warming had a significant negative impact on ACSR in temperate coniferous forests (**Figure 6B**). In deciduous broad-leaved forests, the increase in climate warming led to a significant rise in their ACSR, this resulted in a shift from negative to positive mean values as per simulations (**Figure 6C**).

**Figure 7:**
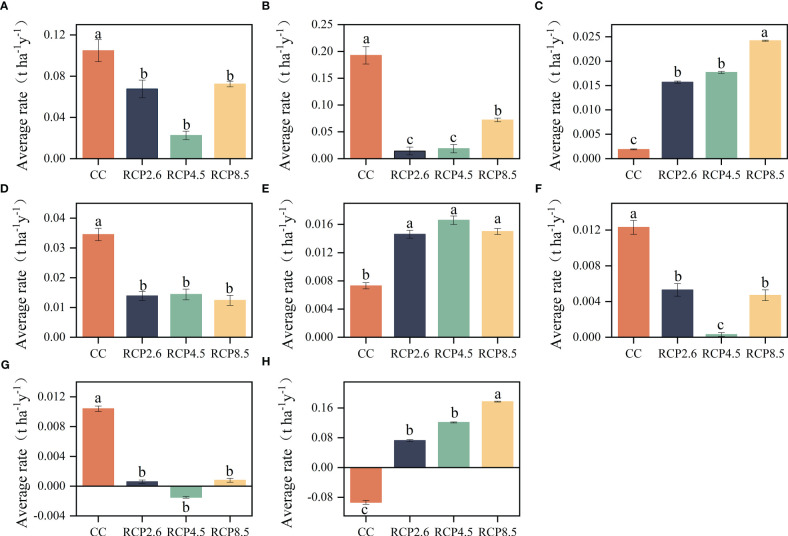
Results of multiple comparisons of the influences on the aboveground carbon sequestration rates major tree species. **(A)** Spruce, **(B)** Fir, **(C)** Chinese hemlock, **(D)** Minjiang cypress, **(E)** Cork oak, **(F)** Locust, **(G)** Chinese toon, **(H)** Birch. Significant differences between different climate scenarios are indicated with different lowercase letters (P < 0.05).

Climate change only significantly affected the ACSR of some tree species (**Supplementary Table 2**). Specifically, the ACSR of spruce was significantly reduced by climate warming, with no discernible difference observed across various warming scenarios (**Figure 7A**). The ACSR of fir was negatively impacted by climate warming, however, it was observed that the effects of RCP8.5 were significantly different from the other two warming scenarios (**Figure 7B**). The Chinese hemlock exhibited a significantly higher ACSR for all warming scenarios compared to the current climate, among the three warming scenarios, the ACSR for RCP8.5 was significantly higher than the other two (**Figure 7C**). The impact of climate warming on Minjiang cypress was found to be similar to that of spruce, the study revealed that warming had significant negative effect on the ACSR of Minjiang cypress (**Figure 7D**). The ACSR of cork oak and birch was significantly increased by climate warming, especially RCP8.5 produced a significant increase in the ACSR of birch (**Figures 7E, H**). ACSR of locust and Chinese toon had significant decreases under warming scenarios (**Figures 7F, G**).

### 3.5 Spatial distribution of carbon stocks

**Figure 8:**
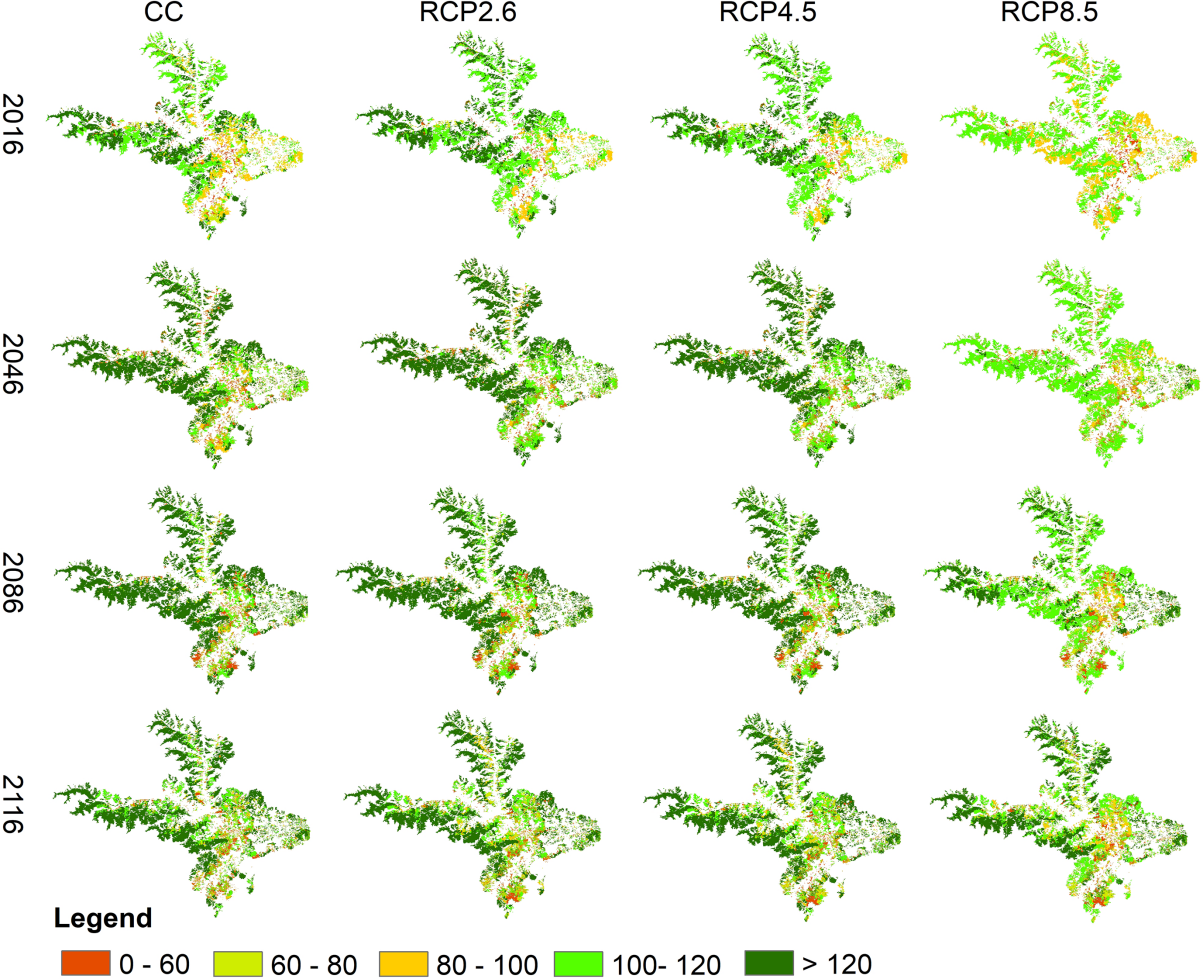
Spatial distribution of forest carbon stocks under different climate scenarios.

Forest carbon stocks showed a large variation in different stages under different climate scenarios (**Figure 8**). Our study found that climate warming had a mixed impact on forest carbon stocks in the area. In the RCP2.6 scenario, there was a positive effect on forest carbon stocks until the 2060s, followed by a slight suppression. Similarly, in the RCP4.5 scenario, there was a positive effect until the 2040s. However, in the RCP8.5 scenario, there was a dramatic warming that noticeably reduced forest carbon stocks. Our study also found that appropriate climate warming promoted carbon stock in the southeastern part of the area, whereas drastic warming had a suppression effect in the high-altitude areas of the western and northeastern regions.

## 4 Discussion

Our study found that climate warming has a significant impact on the ACSR of forests in this transitional ecotone. More specifically, we found that forest ACSR was influenced by various climate change scenarios, forest types, species, and successional stages. The findings of our study suggest that warming has a significant impact on different tree species. In particular, it was observed that warming led to a decrease in the ACSR of coniferous trees, whereas it had a positive effect on the ACSR in broad-leaved trees. These results are consistent with previous research conducted in this field (Ma et al., 2014). The study revealed that warming had a negative impact on the ACSR of spruce and fir, and this negative trend was observed in the later stages of the simulation. We suggest that this may be attributed to the species’ adaptation to current temperature conditions and the inhibitory effect of increased temperature on their photosynthesis. Consequently, warming is expected to exert a stronger negative impact on the ACSR of spruce and fir compared to CO_2_ fertilization, and further warming could continue to inhibit their growth (Coops and Waring, 2011). Consistent with our findings, Liao et al. demonstrated that future warming scenarios would result in a contraction of the habitat range of spruce and fir on the eastern Tibetan Plateau, highlighting their vulnerability to climate warming (Liao et al., 2020). The warming has positively influenced the ACSR of Chinese hemlock, with the RCP8.5 scenario having the most pronounced enhancing effect. This may be attributed to the higher maximum photosynthetic temperature tolerance of Chinese hemlock, the current warming not yet surpassing its threshold, and the temperature increase promoting its photosynthesis, consequently increasing its ACSR. Other coniferous trees, such as Minjiang cypress and Huashan pine, exhibit a similar response to climate warming as spruce and fir. Broad-leaved tree species generally exhibit a positive response in terms of ACSR to climate warming (Cheng and Yan, 2008; Zhang et al., 2023). Research indicates that climate warming is more favorable for broad-leaved trees as compared to subalpine conifers, the latter is experiencing a decline in growth due to standing encroachment (Cao et al., 2021). Climate warming can greatly enhance the ACSR of birch, resulting in a distinct “v” shape in the ACSR change. This is due to the fact that birch, as a pioneer tree species, reaches its natural lifespan and experiences mortality after a short period of increased ACSR, leading to a subsequent decline in ACSR. At the community level, climate warming negatively affected the ACSR of cold temperate coniferous and temperate coniferous forests. In contrast, deciduous broad-leaved forests exhibited a significant increase in ACSR as a result of climate warming. The impact of climate warming on evergreen broad-leaved forests was relatively smaller, which could be attributed to their lower proportion in the region, resulting in a less significant effect.

Our study revealed that climate warming does not alter the trend of ACSR at the landscape scale. After a brief increase, the ACSR continues to decline. This decline may be attributed to artificial plantations established after harvesting in this ecotone. The current forest is in a young state and capable of ongoing carbon sequestration. However, the natural mortality of uniformly planted plantations leads to a significant reduction in ACSR (Wang et al., 2016; Urbano and Keeton, 2017). At the landscape scale, the RCP8.5 scenario has exhibited certain positive effects on ACSR in this ecotone. However, the RCP4.5 and RCP2.6 scenarios have resulted in a decrease in ACSR compared to the current climate. This can be attributed to a notable increase in ACSR of broad-leaved trees resulting from the RCP8.5 scenario. The ACSR of broad-leaved trees has experienced a significant rise due to climate warming. However, under severe climate warming scenarios in the future, the forest carbon stock in the area will be significantly diminished. This is because spruce and fir forests, which represent the dominant communities in our study region, cover the largest area and possess the highest forest carbon sequestration capacity in this ecoregion (Zhang et al., 2013). However, the climate warming scenario has significantly suppressed the ACSR of spruce and fir, leading to a decrease in forest carbon stock.

Our results do not aim to accurately predict the ACSR of this transition ecotone under climate warming scenarios. Instead, the experimental design and model results aim to uncover general patterns of warming effects on carbon sequestration capacity across different ecological scales through controlled experiments. However, the experimental design does not consider certain factors that undoubtedly influence species succession and carbon sequestration capacity, such as increased frequency of fires in warming scenarios, land use changes, and pests and diseases. These limitations indicate a direction for future research. Previous studies have examined the sensitivity and uncertainty of the LANDIS-II model (Gustafson et al., 2010; Thompson et al., 2011; Xiao et al., 2017), revealing that the model input is highly stable. To minimize stochastic error, we conducted repeated simulations and found minimal variability between these repetitions. Our study yields reliable results that highlight the variations in forest ACSR across different scales within the transitional ecotone of the eastern Tibetan Plateau, under different warming scenarios. These findings offer valuable insights for enhancing the forest carbon sequestration capacity in the region.

## 5 Conclusion

In this study, the forest landscape model LANDIS-II was utilized to simulate the effects of climate warming on the ACSR of the eastern Tibetan Plateau Forest transition ecotone. Our results demonstrated that climate warming significantly affects the ACSR in this region. At the landscape scale, it was found that climate warming did not have a significant impact on ACSR, however, the RCP8.5 scenario led to an increase in forest ACSR, whereas the RCP2.6 and RCP4.5 scenarios resulted in a decrease in forest ACSR. The impact of climate warming on ACSR varied in different forest types. In cold temperate coniferous forests and temperate coniferous forests, ACSR was significantly reduced. However, in deciduous broad-leaved forests, ACSR increased significantly due to climate warming. Evergreen broad-leaved forests in the study area were found to be less responsive to climate change. At the scale of tree species, the top communities in our research region, namely spruce and fir, exhibited a significant decrease in ACSR as a result of climate warming. Additionally, climate warming led to a decrease in ACSR of other coniferous trees, with the exception of Chinese hemlock. The response of broad-leaved species’ ACSR to climate warming was mostly positive, particularly in the case of birch, where there was a significant increase in ACSR due to climate warming. In addition, the study found that a moderate increase in temperature had a positive effect on the increase of forest carbon stock in the area, however, a drastic increase in temperature had a significant negative impact on the forest carbon stock in the study area. The findings of this study provide valuable insights into the connection between forest carbon sequestration capacity and climate warming in the eastern Tibetan Plateau. Additionally, these results have practical applications for promoting sustainable forest management in the region in the coming years.

## Data availability statement

The original contributions presented in the study are included in the article/**Supplementary Material**. Further inquiries can be directed to the corresponding author.

## Author contributions

YL: formal analysis, investigation, and writing - original draft. NC: conceptualization, resources, writing – review & editing. JX: conceptualization, methodology, resources, writing – review & editing, and funding acquisition. YK: conceptualization, writing – review & editing, and funding acquisition. YYL, XY, and GQ: formal analysis and validation. CG and YB: investigation and data acquisition. PR: writing – review & editing, and funding acquisition. All authors contributed intellectual input and assistance to this study and manuscript preparation.

## References

[B1] Aber J. D. Federer C. A. (1992). A generalized, lumped-parameter model of photosynthesis, evapotranspiration and net primary production in temperate and boreal forest ecosystems. Oecologia 92, 463–474. doi: 10.1007/BF00317837 28313216

[B2] Bachelot B. Alonso-Rodríguez A. M. Aldrich-Wolfe L. Cavaleri M. A. Reed S. C. Wood T. E. (2020). Altered climate leads to positive density-dependent feedbacks in a tropical wet forest. Global Change Biol. 26, 3417–3428. doi: 10.1111/gcb.15087 32196863

[B3] Cai Q. Ding J. Zhang Z. Hu J. Wang Q. Yin M.. (2019). Distribution patterns and driving factors of leaf c, n and p stoichiometry of coniferous species on the eastern qinghai-xizang plateau, China. Chin. J. Plant Ecol. 43, 1048–1060. doi: 10.17521/cjpe.2019.0221

[B4] Cao J. Liu H. Zhao B. Li Z. Liang B. Shi L.. (2021). High forest stand density exacerbates growth decline of conifers driven by warming but not broad-leaved trees in temperate mixed forest in northeast Asia. Sci. Tot. Environ. 795, 148875. doi: 10.1016/j.scitotenv.2021.148875 34247087

[B5] Chapin F. S. Matson P. A. Mooney H. A. Vitousek P. M. (2002). Principles of terrestrial ecosystem ecology New York: Springer New York. 285–287.

[B6] Chen Q. Xu W. Li S. Fu S. Yan J. (2013). Aboveground biomass and corresponding carbon sequestration ability of four major forest types in south China. Chin. Sci. Bull. 58, 1551–1557. doi: 10.1007/s11434-012-5100-8

[B7] Cheng X. Yan X. (2008). Effects of climate change on typical forest in the northeast of China. Acta Ecol. Sin. 28, 534–543. doi: 10.3321/j.issn:1000-0933.2008.02.011

[B8] Coops N. C. Waring R. H. (2011). Estimating the vulnerability of fifteen tree species under changing climate in Northwest north America. Ecol. Model. 222, 2119–2129. doi: 10.1016/j.ecolmodel.2011.03.033

[B9] Dai E. F. Wu Z. Ge Q. Xi W. Wang X. (2016). Predicting the responses of forest distribution and aboveground biomass to climate change under RCP scenarios in southern China. Global Change Biol. 22, 3642–3661. doi: 10.1111/gcb.13307 27029713

[B10] Dong D. Ni J. (2011). Modeling changes of net primary productivity of karst vegetation in southwestern China using the CASA model. Acta Ecol. Sin. 31, 1855–1866. doi: CNKI:SUN:STXB.0.2011-07-014

[B11] Duan B. Dong T. Zhang X. Zhang Y. Chen J. (2014). Ecophysiological responses of two dominant subalpine tree species betula albo-sinensis and abies faxoniana to intra-and interspecific competition under elevated temperature. For. Ecol. Manage. 323, 20–27. doi: 10.1016/j.foreco.2014.03.036

[B12] Duveneck M. J. Thompson J. R. (2017). Climate change imposes phenological trade-offs on forest net primary productivity. J. Geophys. Res.: Biogeoscie. 122, 2298–2313. doi: 10.1002/2017JG004025

[B13] Fang J. Chen A. Peng C. Zhao S. Ci L. (2001). Changes in forest biomass carbon storage in China between 1949 and 1998. Science 292, 2320–2322. doi: 10.1126/science.1058629 11423660

[B14] Frank D. Reichstein M. Bahn M. Thonicke K. Frank D. Mahecha M. D.. (2015). Effects of climate extremes on the terrestrial carbon cycle: concepts, processes and potential future impacts. Global Change Biol. 21, 2861–2880. doi: 10.1111/gcb.12916 PMC4676934 25752680

[B15] Franks P. J. Adams M. A. Amthor J. S. Barbour M. M. Berry J. A. Ellsworth D. S.. (2013). Sensitivity of plants to changing atmospheric CO 2 concentration: from the geological past to the next century. New Phytol. 197, 1077–1094. doi: 10.1111/nph.12104 23346950

[B16] Frelich L. E. Reich P. B. (2010). Will environmental changes reinforce the impact of global warming on the prairie–forest border of central north America? Front. Ecol. Environ. 8, 371–378. doi: 10.1890/080191

[B17] Gustafson E. J. Miranda B. R. Sturtevant B. R. (2018). Can future CO_2_ concentrations mitigate the negative effects of high temperature and longer droughts on forest growth? Forests 9 (11), 664. doi: 10.3390/f9110664

[B18] Gustafson E. J. Shvidenko A. Z. Sturtevant B. R. Scheller R. M. (2010). Predicting global change effects on forest biomass and composition in south-central Siberia. Ecol. Appl. 20, 700–715. doi: 10.1890/08-1693.1 20437957

[B19] Huo C. Cheng G. Lu X. Fan J. (2010). Simulating the effects of climate change on forest dynamics on gongga mountain, southwest China. J. For. Res. 15, 176–185. doi: 10.1007/s10310-009-0173-1

[B20] IPCC (2013). Climate change 2013: the physical science basis. contribution of working group I to the fifth assessment report of the intergovernmental panel on climate change. climate change Vol. 2013 (New York: Cambridge University Press).

[B21] Kallarackal J. Roby T. (2012). Responses of trees to elevated carbon dioxide and climate change. Biodiversity Conserv. 21, 1327–1342. doi: 10.1007/s10531-012-0254-x

[B22] Liang Y. Gustafson E. J. He H. S. Serra-Diaz J. M. Duveneck M. J. Thompson J. R. (2023). What is the role of disturbance in catalyzing spatial shifts in forest composition and tree species biomass under climate change? Global Change Biol. 29, 1160–1177. doi: 10.1111/gcb.16517 36349470

[B23] Liao Z. Zhang L. Nobis M. P. Wu X. Pan K. Wang K.. (2020). Climate change jointly with migration ability affect future range shifts of dominant fir species in southwest China. Diversity Distribut. 26, 352–367. doi: 10.1111/ddi.13018

[B24] Lindner M. Maroschek M. Netherer S. Kremer A. Barbati A. Garcia-Gonzalo J.. (2010). Climate change impacts, adaptive capacity, and vulnerability of European forest ecosystems. For. Ecol. Manage. 259, 698–709. doi: 10.1016/j.foreco.2009.09.023

[B25] Liu H. Park Williams A. Allen C. D. Guo D. Wu X. Anenkhonov O. A.. (2013). Rapid warming accelerates tree growth decline in semi-arid forests of inner Asia. Global Change Biol. 19, 2500–2510. doi: 10.1111/gcb.12217 23564688

[B26] Liu J. Zou H. X. Bachelot B. Dong T. Zhu Z. Liao Y.. (2021). Predicting the responses of subalpine forest landscape dynamics to climate change on the eastern Tibetan plateau. Global Change Biol. 27, 4352–4366. doi: 10.1111/gcb.15727 PMC13420788 34060175

[B27] Ma J. Hu Y. Bu R. Chang Y. Deng H. Qin Q. (2014). Predicting impacts of climate change on the aboveground carbon sequestration rate of a temperate forest in northeastern China. PloS One 9, e96157. doi: 10.1371/journal.pone.0096157 24763409 PMC3999096

[B28] Miao N. Zhou Z. Shi Z. Feng Q. (2014). Successional dynamics of community structure and species diversity after clear-cutting of faxon fir (Abies faxoniana) forest stands. Acta Ecol. Sin. 34, 3661–3671. doi: 10.5846/stxb201211151605

[B29] Nevins M. T. D'Amato A. W. Foster J. R. (2021). Future forest composition under a changing climate and adaptive forest management in southeastern Vermont, USA. For. Ecol. Manage. 479, 118527. doi: 10.1016/j.foreco.2020.118527

[B30] Piao S.-l. Zhang X. Wang T. Liang E. Wang S. Zhu J.. (2019). Responses and feedback of the Tibetan plateau’s alpine ecosystem to climate change. Chin. Sci. Bull. 64, 2842–2855. doi: 10.1360/TB-2019-0074

[B31] Scheller R. M. Domingo J. B. Sturtevant B. R. Williams J. S. Rudy A. Gustafson E. J.. (2007). Design, development, and application of LANDIS-II, a spatial landscape simulation model with flexible temporal and spatial resolution. Ecol. Model. 201, 409–419. doi: 10.1016/j.ecolmodel.2006.10.009

[B32] Schurman J. S. Babst F. Björklund J. Rydval M. Bače R. Čada V.. (2019). The climatic drivers of primary picea forest growth along the Carpathian arc are changing under rising temperatures. Global Change Biol. 25, 3136–3150. doi: 10.1111/gcb.14721 31166643

[B33] Shifley S. R. Thompson F. R. III Dijak W. D. Fan Z. (2008). Forecasting landscape-scale, cumulative effects of forest management on vegetation and wildlife habitat: a case study of issues, limitations, and opportunities. For. Ecol. Manage. 254, 474–483. doi: 10.1016/j.foreco.2007.08.030

[B34] Sun J. Ye C. C. Liu M. Wang Y. Chen J. Wang S.. (2022). Response of net reduction rate in vegetation carbon uptake to climate change across a unique gradient zone on the Tibetan plateau. Environ. Res. 203, 111894. doi: 10.1016/j.envres.2021.111894 34418448

[B35] Teskey R. Wertin T. Bauweraerts I. Ameye M. McGuire M. A. Steppe K. (2015). Responses of tree species to heat waves and extreme heat events. Plant Cell Environ. 38, 1699–1712. doi: 10.1111/pce.12417 25065257

[B36] Thompson J. R. Foster D. R. Scheller R. Kittredge D. (2011). The influence of land use and climate change on forest biomass and composition in Massachusetts, USA. Ecol. Appl. 21, 2425–2444. doi: 10.1890/10-2383.1 22073633

[B37] Urbano A. R. Keeton W. S. (2017). Carbon dynamics and structural development in recovering secondary forests of the northeastern US. For. Ecol. Manage. 392, 21–35. doi: 10.1016/j.foreco.2017.02.037

[B38] Wang J. Wang G.-X. Wang C.-T. Ran F. Chang R.-Y. (2016). Carbon storage and potentials of the broad-leaved forest in alpine region of the qinghai-xizang plateau, China. Chin. J. Plant Ecol. 40, 374–384. doi: 10.17521/cjpe.2015.0152

[B39] Wang B. Zeng Q. An S. Zhang H. Bai X. (2017). C: N: P stoichiometry characteristics of plants-litter-soils in two kind types of natural secondary forest on the ziwuling region of the loess plateau. Acta Ecol. Sin. 37, 5461–5473. doi: 10.5846/stxb201605150936

[B40] Wei Y. Fang Y. (2013). Spatio-temporal characteristics of global warming in the Tibetan plateau during the last 50 years based on a generalised temperature zone-elevation model. PloS One 8, e60044. doi: 10.1371/journal.pone.0060044 23565182 PMC3615011

[B41] Wu Z. Dai E. F. Wu Z. W. Lin M. Z. (2020). Assessing differences in the response of forest aboveground biomass and composition under climate change in subtropical forest transition zone. Sci. Tot. Environ. 706, 135746. doi: 10.1016/j.scitotenv.2019.135746 31787306

[B42] Xiao J. T. Liang Y. He H. S. Thompson J. R. Wang W. J. Fraser J. S.. (2017). The formulations of site-scale processes affect landscape-scale forest change predictions: a comparison between LANDIS PRO and LANDIS-II forest landscape models. Landscape Ecol. 32, 1347–1363. doi: 10.1007/s10980-016-0442-2

[B43] Xu C. Gertner G. Z. Scheller R. M. (2009). Uncertainties in the response of a forest landscape to global climatic change. Global Change Biol. 15, 116–131. doi: 10.1111/j.1365-2486.2008.01705.x

[B44] Yao Y. T. Piao S. L. Wang T. (2018). Future biomass carbon sequestration capacity of Chinese forests. Sci. Bull. 63, 1108–1117. doi: 10.1016/j.scib.2018.07.015 36658990

[B45] Zhang Y. Gu F. Liu S. Liu Y. Li C. (2013). Variations of carbon stock with forest types in subalpine region of southwestern China. For. Ecol. Manage. 300, 88–95. doi: 10.1016/j.foreco.2012.06.010

[B46] Zhang L. Lu X.-M. Zhu H.-Z. Gao S. Sun J. Zhu H.-F.. (2023). A rapid transition from spruce-fir to pine-broadleaf forests in response to disturbances and climate warming on the southeastern qinghai-Tibet plateau. Plant Diversity. doi: 10.1016/j.pld.2023.03.002 PMC12800768 41541260

[B47] Zhao G. Shao G. (2002). Logging restrictions in China: a turning point for forest sustainability. J. Forestry 100, 34–37. doi: 10.1093/jof/100.4.34

[B48] Zhao M. W. Yue T. X. Zhao N. Sun X. F. Zhang X. Y. (2014). Combining LPJ-GUESS and HASM to simulate the spatial distribution of forest vegetation carbon stock in China. J. Geograph. Sci. 24, 249–268. doi: 10.1007/s11442-014-1086-2

[B49] Zhou R. W. Zhang Y. P. Peng M. C. Jin Y. Q. Song Q. H. (2022). Effects of climate change on the carbon sequestration potential of forest vegetation in yunnan province, southwest China. Forests 13, 306. doi: 10.3390/f13020306

[B50] Zhu X. He H. Liu M. Yu G. Sun X. Gao Y. (2010). Spatio-temporal variation of photosynthetically active radiation in China in recent 50 years. J. Geograph. Sci. 20, 803–817. doi: 10.1007/s11442-010-0812-7

