# Supplementary material for: Projecting future aboveground carbon sequestration rate of alpine forest on the eastern Tibetan Plateau in response to climate change
Source: Front Plant Sci. 2023 Jul 6;14:1212406. doi: 10.3389/fpls.2023.1212406 (PMC10359146; doi:10.3389/fpls.2023.1212406)
Supplement: Supplementary file 1 [file DataSheet_1.docx]

Supplementary Material

Projecting future aboveground carbon sequestration rate of alpine forest on the eastern Tibetan Plateau in response to climate change

**1** **Supplementary Figures and Tables**

**1.1 Supplementary Figures**

**
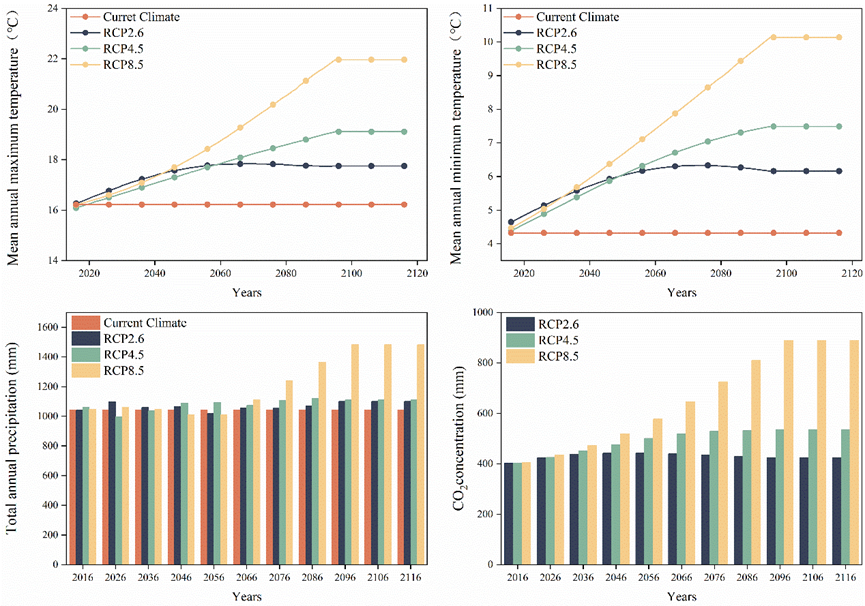
**

**Supplementary Figure 1.** CO2 concentration, temperature and precipitation over the simulation period under current climate and RCP scenarios in the study area.

**
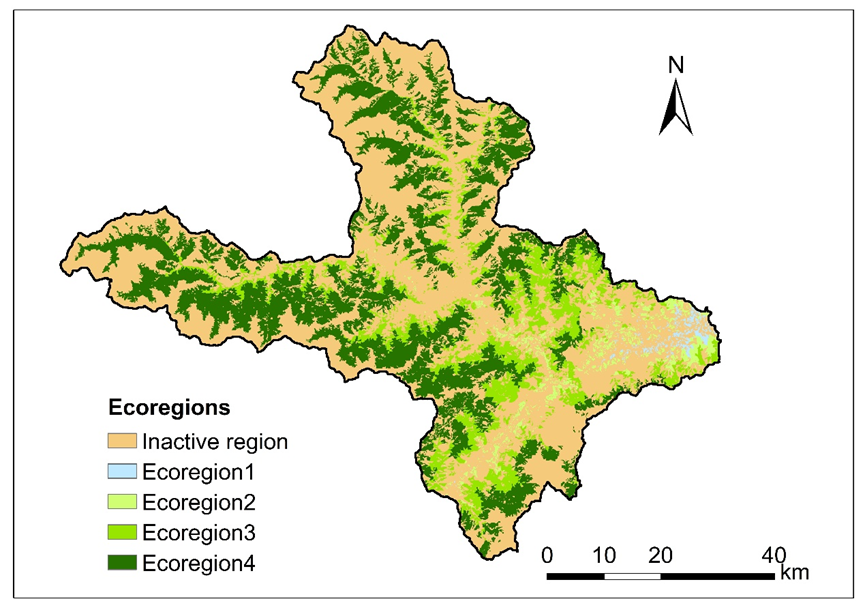
**

**Supplementary Figure 2.** Ecoregions of the study area


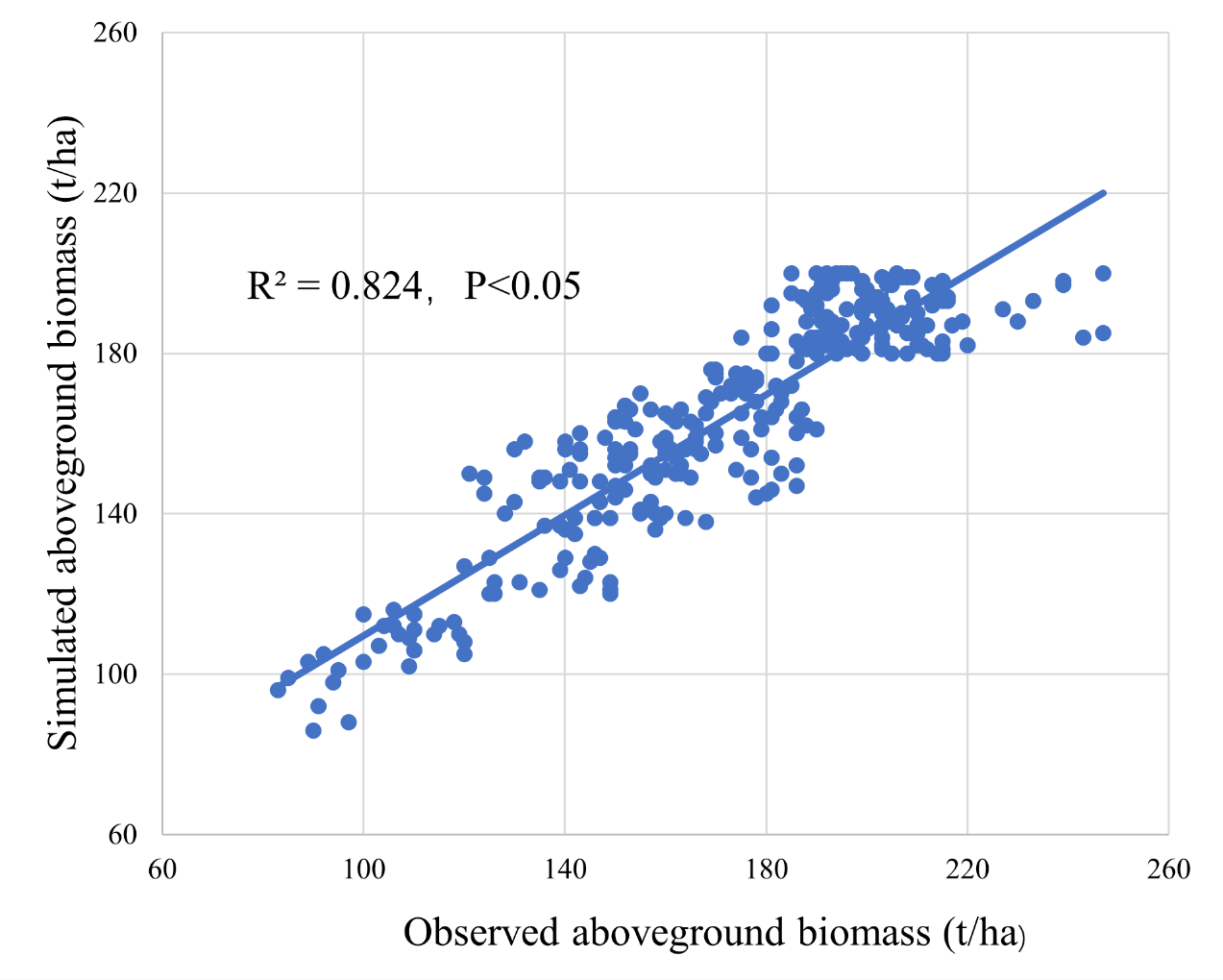


**Supplementary Figure 3.** Comparison between simulated and observed aboveground biomass values in the study area.

**1.2 Supplementary Tables**

**Supplementary Table 1.** Species-specific physiological parameters for 16 tree species on the eastern Tibetan Plateau for PnET-II model.

| Species | FolNCon (%) | FolRet  (% per year) | PsnTMin  (℃) | PsnTOpt  (℃) | SLWmax (g/m^-2^) | AmaxB (umol CO_2_/g leaf/s) |
| --- | --- | --- | --- | --- | --- | --- |
| Spruce | 1.5 | 4 | 0 | 19.36 | 204 | 21.5 |
| Fir | 1.6 | 4 | 0 | 17.81 | 286 | 21.5 |
| Chinese hemlock | 1.2 | 1 | 0 | 23.08 | 170 | 21.5 |
| Huashan pine | 1.5 | 1.6 | 0 | 19.03 | 244 | 21.5 |
| Chinese pine | 1.6 | 2.3 | 4 | 21.67 | 250 | 21.5 |
| Liaodong oak | 2.4 | 1 | 4 | 27.66 | 88 | 71.9 |
| Brown oak | 2.5 | 1 | 4 | 25.05 | 54 | 71.9 |
| Cork oak | 2.3 | 1 | 4 | 26.08 | 88 | 71.9 |
| Ring-cupped oak | 2.7 | 1 | 4 | 25.42 | 79 | 71.9 |
| Minjiang cypress | 1.3 | 2 | 0 | 20.88 | 222 | 71.9 |
| Locust | 2.1 | 1 | 4 | 22.64 | 80 | 71.9 |
| Chinese toon | 2.4 | 1 | 4 | 22.65 | 80 | 71.9 |
| Maple | 2.5 | 1 | 4 | 25.86 | 75 | 71.9 |
| Birch | 2.3 | 1 | 4 | 22.32 | 66 | 71.9 |
| Aspen | 2.5 | 1 | 4 | 22.64 | 80 | 71.9 |
| Alder | 2.1 | 1 | 4 | 18.67 | 100 | 71.9 |

FolNCon: Foliar nitrogen content. FolRet: Maximum relative growth rate for foliage. PsnTMin: Minimum temperature for photosynthesis. PsnTOpt: Maximum temperature for photosynthesis. SLWmax: Species leaf weight at top canopy. AmaxB: Slope of Amax versus N relationship

**Supplementary Table 2.** Results of multiple comparisons of the influences on all species aboveground carbon sequestration rate.

| Species | *P* | CC | RCP2.6 | RCP4.5 | RCP8.5 |
| --- | --- | --- | --- | --- | --- |
| Spruce | 0.001 | 0.1048a | 0.0676ab | 0.0225b | 0.0724b |
| Fir | <0.01 | 0.1927a | 0.0143c | 0.0186c | 0.0721b |
| Chinese hemlock | <0.01 | 0.0019c | 0.0157b | 0.0177b | 0.0242a |
| Huashan pine | 0.126 | 0.0225 | -0.0020 | -0.0015 | 0.0097 |
| Chinese pine | 0.372 | 0.0019 | -0.0008 | -0.0008 | -0.0006 |
| Liaodong oak | 0.142 | -0.0039 | 0.0018 | 0.0042 | 0.0082 |
| Brown oak | 0.205 | -0.0163397 | -0.0131 | -0.0187 | -0.0188 |
| Cork oak | <0.01 | 0.0073b | 0.0146a | 0.0166a | 0.0150a |
| Ring-cupped oak | 1.00 | -0.0278 | -0.0292 | -0.0291 | -0.0299 |
| Minjiang cypress | <0.01 | 0.0345a | 0.0139b | 0.0144b | 0.0124b |
| Locust | <0.01 | 0.0123a | 0.0053b | 0.0003c | 0.0047b |
| Chinese toon | <0.01 | 0.0104a | 0.0006b | -0.0015b | 0.0008b |
| Maple | 0.573 | 0.0070 | 0.0081 | 0.0095 | 0.0113 |
| Birch | <0.01 | -0.0935c | 0.0725b | 0.1212b | 0.1771a |
| Aspen | <0.01 | 0.0195a | 0.0107b | 0.0036c | -5.53E-05d |
| Alder | 0.298 | -0.0004 | -0.0027 | -0.0026 | -0.0013 |
